# Bilateral adrenal-renal fusion: A radiological diagnosis

**DOI:** 10.1259/bjrcr.20180108

**Published:** 2018-12-10

**Authors:** Rebecca Bamford, Josephine Bretherton, Nicola Rosenfelder, James Bell

**Affiliations:** Royal Free NHS Foundation Trust, London, UK

## Abstract

In normal anatomy, the kidneys and adrenal glands are contained within the renal fascia and separated by a connective tissue capsule derived from mesenchymal tissue. Incomplete encapsulation can occur during embryonic development, resulting in adrenal-renal fusion. The true incidence of this developmental anomaly is unknown, as it has primarily been described in the literature following incidental detection on surgical or histological examination. We report the first documented case of bilateral adrenal-renal fusion, diagnosed radiologically.

## Case report

A 55-year-old male with a past medical history of human immunodeficiency virus presented with increased urinary frequency and haematuria. Physical examination revealed no abdominal tenderness or masses. He was thought to have prostatism and was referred for a pelvic MRI, which showed thickening of the bladder wall. Cystoscopy and bladder biopsy confirmed the presence of a mostly papillary but partly solid high grade (Grade 3) urothelial carcinoma, (pT2 at least) with further prostatic urethral biopsies showing invasive urothelial carcinoma (pT1 at least).

**Figure 1.**
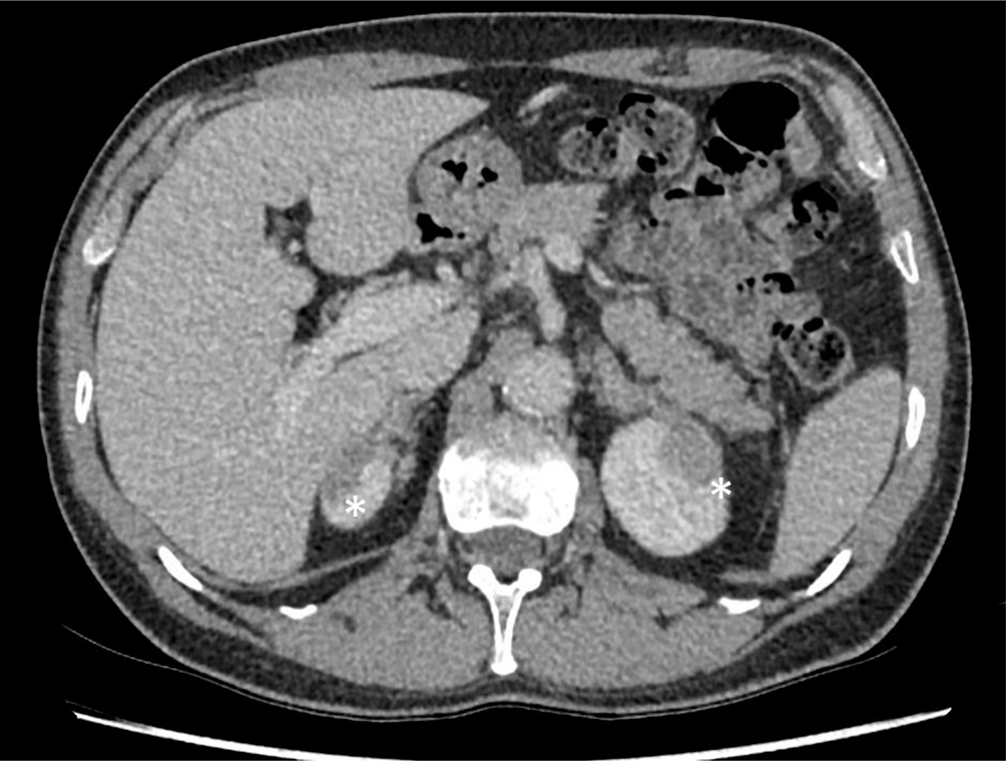
Axial and contrast-enhanced CT image shows bilateral symmetrical well-defined low-attenuation lesions (white asterisks) within the upper poles of both kidneys, inseparable from the adrenal glands and with no visible fat plane between the upper pole of the kidney and the lateral limb of the adrenal gland.

**Figure 2.**
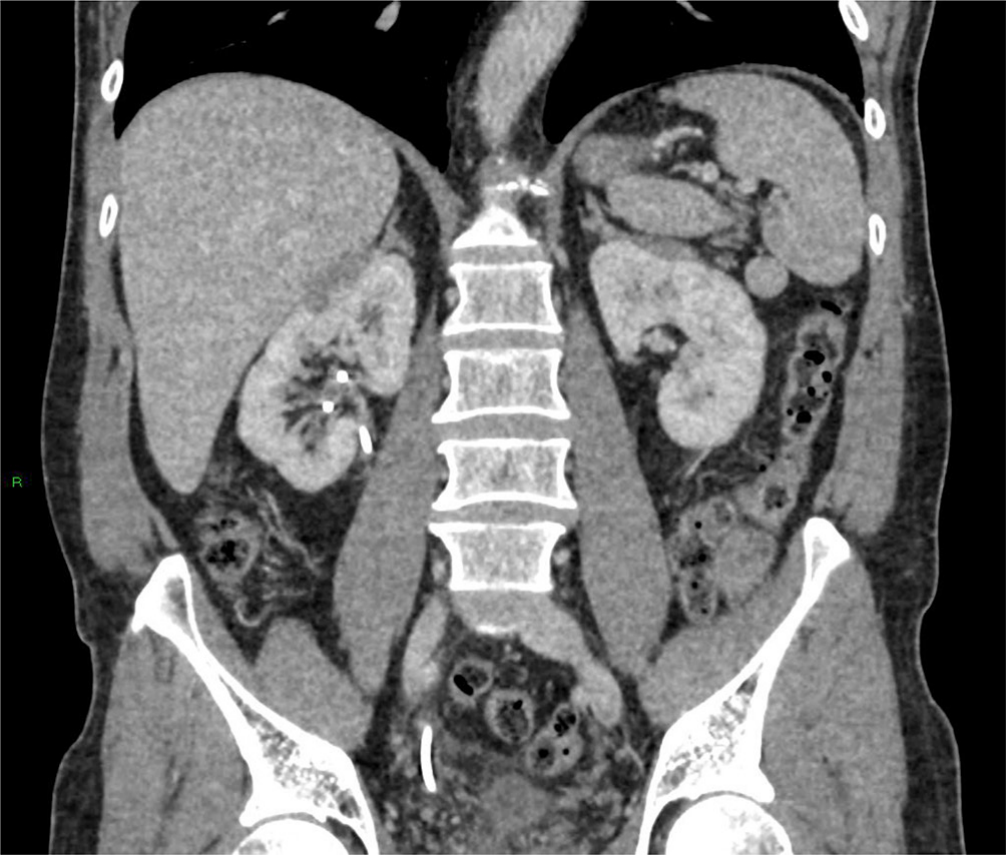
Coronal reformats confirm lack of fat plane between kidney and adrenal gland bilaterally.

**Figure 3.**
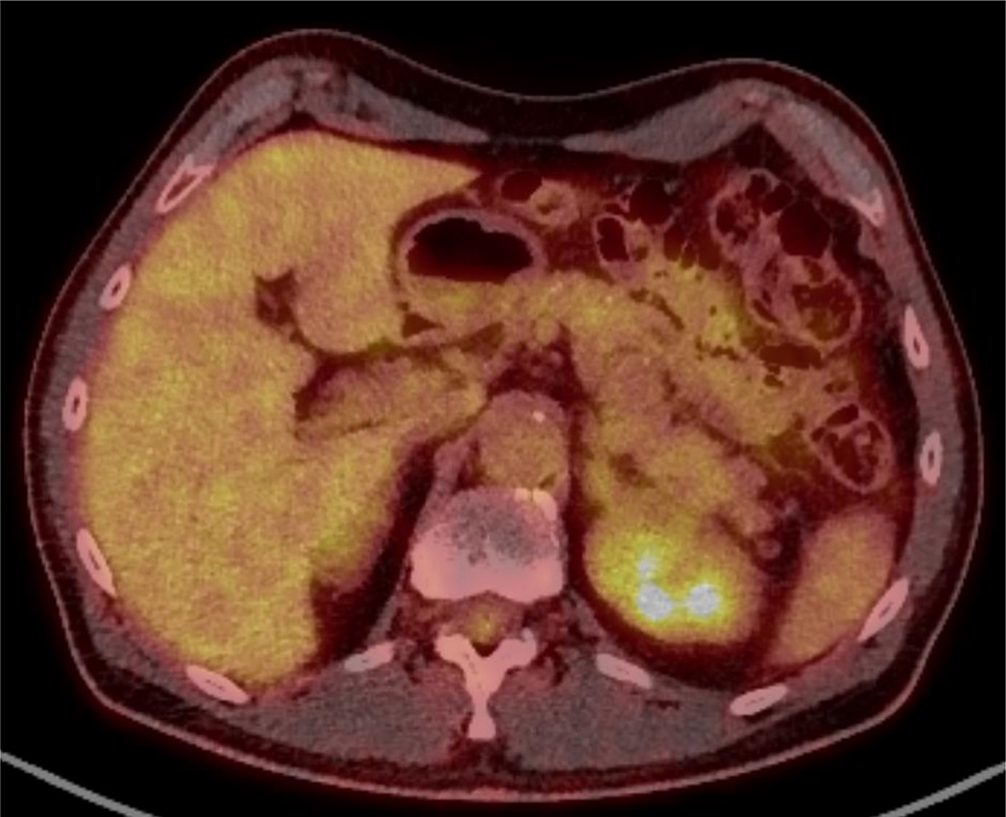
Axial FDG PET CT image through the relevant area demonstrates no significant increased avidity.

A contrast-enhanced staging CT scan was performed using a Toshiba Aquilion scanner, with arterial phase acquisition through the chest and portal venous phase acquisition through the abdomen and pelvis. The only abnormality noted was a nodular appearance of the adrenal glands and symmetrical well-defined low-attenuation subcapsular lesions (mean Hounsfield unit 55) at the upper poles of both kidneys, each measuring 2.3 cm (Figure 1). On review of the multiplanar image reformats, there was no demonstrable fat plane between the renal lesions and the lateral limbs of the adrenal glands (Figure 2). These appearances were thought to represent incidental congenital adrenal-renal fusion. An fludeoxyglucose (FDG) positron emission tomography (PET)-CT scan was performed to exclude adrenal metastases and showed low-grade tracer uptake within the body of the adrenal glands bilaterally in keeping with adrenal hyperplasia but no abnormal uptake to suggest malignancy (Figure 3). Increased tracer uptake was present in a right external iliac lymph node suggestive of metastasis. No other developmental abnormality was detected.

The patient underwent neo-adjuvant chemotherapy, followed by radical cysto-prostatectomy and lymph node clearance for his bladder cancer. A repeat FDG PET-CT performed 5 weeks later following neo-adjuvant chemotherapy showed indeterminate low-grade uptake in the right iliac lymph nodes and stable appearances of the adrenal glands, providing further reassurance that this did indeed represent a benign anatomical variant.

It was not deemed ethical to obtain a tissue sample as the imaging appearances were felt to be classical and the patient will undergo regular surveillance imaging following his bladder cancer treatment.

## Discussion

The adrenal gland is formed of two embryologically-distinct layers: the cortex and medulla. The adrenal cortex is derived from mesoderm and the medulla originates from neural crest cells. Around 52 days post-conception, the adrenal gland begins to separate from the mesenchymal cells and becomes encapsulated by fibrous tissue.^1^ Adrenal-renal fusion was first described by Rokitansky in 1855,^2^ who differentiated between congenital lesions and those acquired following inflammation of the peri-renal fat. The congenital form is considered to be caused by failure of the retroperitoneal mesenchymal cells to stimulate capsule formation.^3^ Consequently, capsule formation is incomplete, and the adrenal gland and kidney become conjoined. The aetiology of adrenal-renal fusion is not known, but it is thought to be a local event around 52 days post-conception, due to the absence of other associated congenital abnormalities.^4^

While isolated adrenal-renal fusion is a benign diagnosis with no clinical implications, there are several case reports in which the radiological appearances have been misinterpreted, particularly when there is a concurrent adrenal lesion, and have resulted in unnecessary surgery. In 2004, Fan et al reported, an intra-operative frozen-section misdiagnosed histologically as a renal cystic mass, leading to a radical nephrectomy. Post-operatively, when the en bloc specimen was reviewed, adrenal-renal fusion and adrenal adenoma were diagnosed.^5^ In 2009, Mahadevia et al described a case of an adrenal adenoma with adrenal-renal fusion which was misdiagnosed as invasive renal cell carcinoma, resulting in an adrenalectomy and partial nephrectomy.^6^ The only previous case study not to have been diagnosed histologically was the intra-operative diagnosis by Boll et al based on the lack of a vascular plane between the renal and adrenal capsule,^7^ which negated the need for a larger resection.

Adrenal-renal fusion is considered difficult to diagnose radiologically, and the imaging appearances described in the existing literature have been observed retrospectively. The characteristic findings are lack of a discrete fat plane between the upper pole of the kidney and adrenal gland, with or without a contiguous well-defined lesion within the adjacent kidney. The routine use of thin-slice CT image acquisition and multiplanar reformats in the last 10–15 years allows better detection of an absent fat plane, although this finding is not specific for adrenal-renal fusion and in unilateral cases it is difficult to exclude an invasive renal, adrenal or retroperitoneal lesion. Furthermore, artefactual degradation of images from diaphragmatic motion can limit evaluation of the peri-renal region. In this particular case, the striking symmetry of the appearances, well-defined margins of the sub capsular renal lesions and lack of suspicious uptake on PET-CT allowed a confident radiological diagnosis to be made without the need for invasive tissue sampling. We are not aware of any published description of the MRI appearances of adrenal-renal fusion, and this could be an interesting addition to the literature; however, MRI was not performed in this case as it would have been purely for academic purposes.

## Learning points

- Adrenal-renal fusion is a clinically insignificant congenital anomaly of unknown incidence, which can be incorrectly characterised as renal or adrenal malignancy.
- The characteristic appearance of adrenal-renal fusion is lack of an intervening fat plane, which can be confirmed on three-dimensional image reformats; however, this is non-specific.
- Reassuring features are symmetry (if bilateral, as in this case), well-defined margins and lack of tracer avidity on PET-CT.
- Correctly identifying this condition could save patients from unnecessary intervention.

## Conclusion

We believe this is the first reported case of bilateral adrenal-renal fusion diagnosed on imaging, and adds to the existing case reports on the condition. We re-iterate the importance of radiologists being aware of this condition and communicating with the operating surgeon if any renal or adrenal surgery is planned.
